# Moving towards high-dose primaquine or single-dose tafenoquine for *Plasmodium vivax* treatment in Cambodia: a meeting report from dissemination of results of the EFFORT trial to stakeholders

**DOI:** 10.1186/s12936-025-05691-1

**Published:** 2026-01-03

**Authors:** Lek Dysoley, Sarah Cassidy-Seyoum, Bipin Adhikari, Aaryan Dahal, Nead Phoumen, Sin Srey, Touch Phalla, Prak Vonn, But Engrkruy, Oum Sovanarath, Hor Mengkea, Pov Rathana, Iv Makara, Pen Kim Heng, Yim Heng, Huot Khlok, Khourn Pong, Tov Moeng, Sam Vantha, Ung Soviet, Rin Ravuth, Voeurng Bunreth, Keo Vannak, Ma Sokhann, Ke Kimmen, Khoy Dy, Siv Samol, Koung Lo, Nuon Sokunthea, Hok Kry, Ly Kanha, Leng Naren, Sarith Oum, Yok Sovann, Ros Channavuth, Chhun Bunmeng, Jayme Hughes, Hannah Brindle, Yang Hu, Sovann Peng, Prum Mardi, Im Chanry, Sodavuth Preap, Socheat Heoy, Farooq Sabawoon, Thoang Sokha, Pascal Ringwald, Caroline A. Lynch, Ric N. Price, Rupam Tripura, Lorenz von Seidlein, Angela Devine, Kamala Thriemer

**Affiliations:** 1 National Center for Parasitology, Entomology, and Malaria Control, Phnom Penh, Cambodia; 2 School of Public Health, National Institute of Public Health, Phnom Penh, Cambodia; 3 Global and Tropical Health Division, Menzies School of Health Research, Charles Darwin University, Darwin, Australia; 4 Mahidol Oxford Research Unit, Faculty of Tropical Medicine, Mahidol University, Bangkok, Thailand; 5 Center for Tropical Medicine and Global Health, Nuffield Department of Medicine, University of Oxford, Oxford, UK; 6 Provincial Health Department, Kampong Cham, Cambodia; 7 Provincial Health Department, Kampong Thom, Cambodia; 8 Provincial Health Department, Kampong Chhnang, Cambodia; 9 Provincial Health Department, Sisophon, Banteay Meanchey Cambodia; 10 Provincial Health Department, Krong Ban Lung, Ratanakiri Cambodia; 11 Provincial Health Department, Preah Sihanouk, Cambodia; 12 Provincial Health Department, Siem Reap, Cambodia; 13 Provincial Health Department, Senmonorom, Mondulkiri Cambodia; 14 Provincial Health Department, Kampot, Cambodia; 15 Provincial Health Department, Samraong, Oddar Meanchey Cambodia; 16 Provincial Health Department, Koh Kong, Cambodia; 17 Provincial Health Department, Steung Treng, Cambodia; 18 Provincial Health Department, Battambang, Cambodia; 19 Provincial Health Department, Kandaol Chrum, Tbong Khmum Cambodia; 20 Provincial Health Department, Pursat, Cambodia; 21 Provincial Health Department, Doun Kaev, Takeo Cambodia; 22 Provincial Health Department, Preah Vihear, Cambodia; 23 Provincial Health Department, Chbar Mon, Kampong Speu Cambodia; 24 Provincial Health Department, Kratie, Cambodia; 25 Provincial Health Department, Pailin, Cambodia; 26 Provincial Health Department, Prey Veng, Cambodia; 27 Clinton Health Access Initiative, Phnom Penh, Cambodia; 28 Catholic Services Relief, Phnom Penh, Cambodia; 29 USAID Global Health Supply Chain Program-Procurement and Supply Management, Phnom Penh, Cambodia; 30 Abt Global, Phnom Penh, Cambodia; 31 UNOPS, Phnom Penh, Cambodia; 32 Malaria Consortium, Phnom Penh, Cambodia; 33 World Health Organization, Mekong Malaria Elimination Programme, Phnom Penh, Cambodia; 34 MMV Medicines for Malaria Venture, Geneva, Switzerland; 35 Centre for Health Policy, Melbourne School of Population and Global Health, University of Melbourne, Melbourne, Australia

**Keywords:** Cambodia, Malaria, Vivax, Radical cure, Tafenoquine, Primaquine

## Abstract

Cambodia has targeted malaria elimination by 2025. As the malaria burden has decreased in Cambodia, transmission has become more focal, and *Plasmodium vivax* has become the predominant species. The recurrent nature of *P. vivax*, due to its dormant liver stages causing relapses, is the main obstacle to malaria elimination in Cambodia. In 2021, Cambodia’s National Center for Parasitology, Entomology and Malaria Control (CNM) rolled out low-dose 14-day primaquine (total dose 3.5 mg/kg) supported by point-of-care quantitative testing for glucose-6-phosphate dehydrogenase deficiency. However, this treatment is limited by poor adherence to its prolonged duration and suboptimal efficacy of the low total dose. The EFFORT clinical trial was conducted in four malaria-endemic countries, including Cambodia, to assess the safety and effectiveness of a 7-day unsupervised high-dose course of primaquine (7 mg/kg total dose) and single dose tafenoquine (300 mg) compared to 14-day unsupervised low-dose primaquine for the treatment of patients presenting with *P. vivax* malaria. In addition, data were collected on the feasibility and cost-effectiveness of these treatment options. CNM organized the national dissemination of the EFFORT study results on March 27, 2025, to inform key stakeholders and discuss the implications of the study findings for policy and practice in Cambodia.

## Background

Cambodia has made significant progress in reducing its malaria burden in the last 30 years [1, 2]. Between 2004 and 2014, reported malaria cases dropped by more than half from 113,855 to 56,271 cases [2]. In 2015, as part of the Greater Mekong Subregion’s (GMS) efforts to combat *Plasmodium falciparum* resistance, Cambodia committed to malaria elimination by 2030 [3]. In 2016, Cambodia set a national malaria elimination target of 2025 and established a National Malaria Elimination Framework [2], which was updated in 2021 [4]. As a result of the strategies outlined in these elimination frameworks, significant reductions in burden have continued. Between 2020 and 2022, confirmed malaria cases decreased from 9490 to 4053 [5, 6]. As case numbers have decreased, malaria transmission in Cambodia has become focalized [7], and *Plasmodium vivax* has become the predominant species [2, 4, 8]. In 2023, there were 1384 reported cases, of which 95% were *P. vivax* [8]. Hence, *P. vivax* has become the main hurdle for malaria elimination.

*Plasmodium vivax* forms dormant liver stage parasites, hypnozoites, which can be reactivated weeks to months after initial infection (relapse), causing acute disease [9, 10]. To clear the hypnozoite stage of the parasite, 8-aminoquinolines are used. The only available 8-aminoquinolines are primaquine and, more recently, tafenoquine. Both drugs can cause haemolysis in individuals with a deficiency in the enzyme glucose-6-phosphate dehydrogenase (G6PD) [11]. The prevalence of severe G6PD deficiency reaches 20% in some areas of Cambodia [12–15]. To reduce the risk of drug-induced hemolysis, a low daily dose (0.25 mg/kg/day) of primaquine treatment administered over 14 days (total dose 3.5 mg/kg) was recommended [16, 17]. This recommendation was not implemented until 2021, when the Cambodian National Center for Parasitology, Entomology and Malaria Control (CNM) rolled out the STANDARD G6PD^®^ test (SD Biosensor, Republic of Korea) to identify patients with G6PD deficiency, allowing for a safer administration of primaquine [18, 19]. However, the 14-day primaquine regimen has limited effectiveness because of poor adherence to the prolonged treatment regimen [20–22]. To address this challenge, the World Health Organization (WHO) recommended providing the same total dose (3.5 mg/kg) over 7 days (0.5/mg/day). CNM updated their national treatment guidelines accordingly and implemented this regimen in 2023 [23]. Recent evidence, however, suggests that the low total dose of primaquine has sub-optimal anti-relapse efficacy in almost all geographic areas, and a higher total dose halves the risk of recurrence [24]. Therefore, in 2024, the WHO recommended a high-dose treatment (7 mg/kg total dose) administered over 14 or 7 days [25], but this guidance has not yet been implemented in most malaria-endemic regions.

Tafenoquine, now licensed in 10 malaria endemic countries [26, 27], is a single dose treatment overcoming the adherence issues inherent to primaquine treatment. Tafenoquine was tested in comparison with low-dose primaquine [28] and is restricted to use with chloroquine, as schizonticidal treatment [29, 30]. This restriction was implemented following results of a clinical trial, in which tafenoquine was prescribed in combination with dihydroartemisinin-piperaquine (DHA-piperaquine) in Papua, Indonesia, which resulted in very low efficacy, interpreted as being due to a drug-drug interaction between tafenoquine and artemisinin combination therapies (ACT) [31].

The comparative effectiveness of tafenoquine and high-dose primaquine when unsupervised was unknown. The EFFORT study (NCT04411836) assessed the effectiveness and safety of unsupervised 7-day high-dose primaquine (1 mg/kg/day, 7 mg/kg total dose) and a single dose of tafenoquine (300 mg) compared to the 14-day low-dose primaquine treatment (0.25 mg/kg/day, 3.5 mg/kg total dose). The study was conducted as a multi-centre randomized controlled trial with study sites in Cambodia, Ethiopia, Indonesia, and Pakistan. In Ethiopia and Pakistan, primaquine and tafenoquine were administered with chloroquine as the schizontocidal drug, in Cambodia with artesunate-pyronaridine and in Indonesia with DHA-piperaquine.

Detailed findings of the trial are presented elsewhere [32]. In brief, the trial showed that the 7-day high-dose primaquine and tafenoquine regimens were well tolerated, and both treatments led to a substantial reduction of recurrences compared to patients treated with 14-day low-dose primaquine. In Cambodia, where tafenoquine was administered with artesunate-pyronaridine, the risk of recurrence was 15.6% at 6 months, lower than both the low- and high-dose primaquine arms (27.3% and 18.4%, respectively) and substantially lower than the anticipated background risk of recurrence of around 80% [33]. These findings challenge the assumption that tafenoquine and ACT cannot prevent relapse [31, 34, 35].

Implementing tafenoquine or high-dose primaquine requires G6PD testing [36, 37]. In Cambodia, G6PD testing is conducted at health centres; however, qualitative data collected alongside the trial suggested that the implementation of G6PD testing at the community level is feasible, and this could reduce the number of patients requiring referral to the health centre for G6PD testing and treatment [18, 38–40]. Feasibility and acceptability data also indicated that implementing either 7-day high-dose primaquine or single-dose tafenoquine would require enhanced monitoring to ensure safe delivery, which needs to be tailored to the country context [41].

Health economic analyses are ongoing, but the preliminary cost-effectiveness analysis found that 7-day high-dose primaquine and tafenoquine were likely to reduce costs as compared to 14-day low-dose primaquine in most study settings due to the reduction in malaria cases [42]. This is particularly true in settings like Cambodia, where G6PD screening and routine follow-up visits are already part of routine clinical practice. Another key finding from the preliminary analyses was that the EuroQoL 5 dimensions – 5L (EQ-5D-5L) survey, a standardized instrument used to measure health-related quality of life, was sensitive enough to capture differences in quality of life from enrollment to 3 weeks post treatment [42].

### Meeting description

Organized by CNM in collaboration with Menzies School of Health Research and Mahidol-Oxford Tropical Medicine Research Unit (MORU), the dissemination of EFFORT study results in Cambodia took place on March 27, 2025. The objective of the meeting was to disseminate the results of the EFFORT study among key stakeholders and discuss implications for the case management of *P. vivax* malaria in Cambodia. Participants included CNM officials, sub-national malaria officers, and implementation and research partners. Subnational program staff in attendance included 21 provincial malaria supervisors and 15 operational district malaria supervisors. Fifteen partner representatives attended the meeting, among them were the WHO, United Nations Office for Project Services (UNOPS), Clinton Health Access Initiative (CHAI), President Malaria Initiative (PMI), Catholic Relief Services (CRS), and MMV Medicine for Malaria Venture (MMV). In total, 51 people attended the meeting.

The agenda included a presentation of the effectiveness and safety data from the trial, including country-specific trial data, as well as results from the feasibility assessments and health economic components of the study. Each thematic presentation was followed by question-and-answer sessions. The question-and-answer session on health economics was designed to inform further analysis and included interactive questions using Mentimeter (Table 1).

**Table 1:** Questions asked to inform future health economic analyses

Question	Answer options
Which comparators are important for the health economic analysis?	• TQ vs low-dose PQ • High-dose PQ vs low-dose PQ • TQ vs high-dose PQ • TQ vs high-dose PQ vs low-dose PQ
What outcomes are important to inform policy making?	• Costs only • Cost per cases averted • Cost per QALY gained • Cost per DALY averted • Does not matter for decision making
What type of model framework would be most helpful to better inform policy decisions?	• Transmission model • Decision tree (no transmission) • Does not matter
Are treatment options for falciparum malaria still relevant for decision making?	• Yes – assess independently of *P.v* • Yes – would consider same treatment for *P.v.* and *P.f* • No
Is there anything else that would be helpful to see for decision making?	[free text responses]

*DALY* disability-adjusted life-year, *P.f.* *Plasmodium falciparum, P.v.* *Plasmodium vivax*, *PQ* primaquine, *QALY* quality-adjusted life-year, *TQ* tafenoquine

After lunch, attendees were split into three groups for roundtable discussions. The application of study results to the current malaria situation in Cambodia was addressed with two questions: (i) What do the EFFORT results mean for *P. vivax* case management in Cambodia? (ii) What do they mean for the prevention of reintroduction strategies?

Throughout the meeting, presentations and discussions were documented in real time by the authors (SCS, BA and AD) and later collated by SCS to distil key themes. The following identified themes are presented: (i) demand for both 7-day high-dose primaquine and tafenoquine with a preference for tafenoquine, (ii) the need for flexible and all-encompassing national treatment guidelines, (iii) the applicability of study results to outbreak and re-introduction responses, and (iv) the use of health economic data as part of decision making.

### Preference for tafenoquine

All participants agreed that treatment guidelines should be updated in line with the EFFORT study findings, given their local applicability. The added value of 7-day high-dose primaquine and single-dose tafenoquine was recognized while participants emphasized that tafenoquine was especially useful from a programmatic perspective to improve adherence. These preferences were in line with findings from a recent study in Cambodia, assessing the acceptability of these treatment options among policymakers, sub-national program staff, health facility workers, village malaria workers, mobile malaria workers, and patients [43].

While there is demand for 7-day high-dose primaquine and single-dose tafenoquine, several factors, including the need for G6PD testing, treatment monitoring, setting up a pharmacovigilance structure, and the potential off-label use of tafenoquine with an artemisinin-based combination, were emphasized as considerations for policy change [36, 41]. The implementation of quantitative G6PD testing in Cambodia in 2021 is likely to facilitate the rollout of 7-day high-dose primaquine or single-dose tafenoquine in Cambodia, while current treatment monitoring guidelines could be adapted to ensure patient safety [41, 44]. Strengthening the pharmacovigilance structure was identified at the meeting as a key consideration requiring further work.

An important consideration in Cambodia is the current restriction for the exclusive use of tafenoquine with chloroquine [29] and the current WHO recommendation limiting its use to South America [37]. The first restriction is based on the results of a trial conducted in Indonesia (the INSPECTOR study), which showed low efficacy of tafenoquine (300 mg) when combined with DHA-piperaquine [31]. As a result, tafenoquine use for radical cure is precluded from 14 of the 41 *P. vivax*-endemic countries, including Cambodia, where artemisinin-based combinations are the first-line treatment [45]. Drug-drug interactions have been proposed as an explanation for the poor efficacy in INSPECTOR, but there are other plausible explanations, including under-dosing of tafenoquine and the high hypnozoite burden in the study setting in Papua, Indonesia, and strain-specific characteristics [31, 34]. The EFFORT results, particularly the Cambodia data, challenge these restrictions [32].

In Cambodia, the first-line schizontocidal treatment is artemisinin-mefloquine (ASMQ), with plans to transition to artesunate-pyronaridine by the end of 2025. Therefore, the use of tafenoquine in Cambodia would either require switching to chloroquine as a first-line treatment for *P. vivax* malaria or using tafenoquine off-label, the latter raising potential accountability and responsibility issues for CNM. Based on the discussions at the meeting, there was a lot of interest in the use of tafenoquine, but its off-label use would come with major challenges, including procurement, requiring innovative and potentially regional procurement systems and discussions with regulatory bodies.

### Need for flexible and broad treatment recommendations

The first step in implementing either tafenoquine or 7-day high-dose primaquine is to amend Cambodia’s national treatment guidelines. Meeting discussions highlighted the need for flexibility in guidelines. Flexible guidelines were characterized by the inclusion of all potential treatment options so that they could be operationalized once feasibility constraints, including procurement and training, are addressed. This approach is especially relevant as it is unlikely that updates to the national treatment guidelines after 2025 will be frequent, given that the country is so close to malaria elimination [4].

Changing national malaria treatment policies is a challenging process [46–48]. National policymakers have to consider input from several global and national stakeholders, including the WHO, national regulatory bodies, researchers, and both national and international funders, in addition to requiring significant financial and human resources. Increased country ownership, risk acceptance, process and system innovations, as well as better planning and readiness for delivery, have been suggested to improve the policymaking process [46]. Many of these suggestions have already been implemented in Cambodia, with policy changes to malaria guidelines being relatively frequent and driven by global and local evidence generation [43]. However, when such process improvements are limited, flexible guidelines could be an alternative mechanism to support the accelerated implementation of new treatment options.

Guidelines including multiple treatment options could also provide opportunities to tailor treatment selection at the provincial or district level depending on their unique circumstances, e.g., case burden and capacity for pharmacovigilance. With more choice, however, comes the need for clear operational guidelines and training of healthcare providers to ensure they understand the rationale and use of treatment regimens and considerations with their administration to patients. Already inherent in the use of 7-day high-dose primaquine or single-dose tafenoquine is the need to administer an alternative treatment for those with G6PD levels under 70% activity and, therefore, complex treatment algorithms. In Brazil, where tafenoquine has been piloted, a tiered treatment algorithm was implemented and operationally feasible [49]. Tafenoquine was provided to patients with G6PD activity ≥ 70%, low-dose 7-day primaquine (0.5 mg/kg/day) to those with activities between 30 and 70%, and 8-week (0.75 mg/week) primaquine to G6PD deficient patients [49, 50]. The operational feasibility of such a treatment algorithm in Cambodia, where the administration of 8-week primaquine for G6PD deficient individuals has only recently commenced, is yet to be determined.

### Applicability to outbreak responses and reintroduction

A key point of discussion was the applicability of study results in the current context of malaria burden and transmission in Cambodia. At the time of the meeting, only eight malaria cases had been reported in 2025. Though case numbers were expected to increase during the rainy season between June and September, such a low burden prompted concerns about the cost-effectiveness of changing to a new treatment, given the resources required to implement those changes. Participants, however, attributed added value to these shorter treatment options, especially tafenoquine, in the context of the remaining high-risk populations, outbreak responses, and the prevention of reintroduction in the future.

The remaining patients diagnosed with malaria are mostly from mobile migrant populations who cross international borders. Treatment uptake and adherence among these populations can be difficult [18, 44]. The availability of a single-dose option would most likely increase uptake and remove the adherence barrier. In the event of malaria outbreaks, tafenoquine was considered useful as part of ongoing targeted drug administration campaigns by the national malaria program [51]. Providing tafenoquine to G6PD normal individuals in this context would contribute to reducing the hypnozoite reservoir and, therefore, reducing future onward transmission. Using tafenoquine in this capacity is also being explored in other GMS countries where *P. vivax* is the prevalent malaria species [52, 53]. In the context of obtaining malaria elimination certification and subsequently preventing reintroduction in Cambodia, tafenoquine was considered the preferred option, and this is likely relevant for countries across the GMS; however, this requires sustaining G6PD testing capacity.

### Costs and cost-effectiveness analysis

Meeting attendees were briefed on three health economics aims within the EFFORT trial: (i) estimating total costs from the healthcare provider, household, and societal perspectives, (ii) evaluating whether the EQ-5D survey was sensitive enough to capture changes in quality of life in *P. vivax* malaria patients, and (iii) evaluating the cost-effectiveness of 7-day high-dose primaquine and single-dose tafenoquine compared to 14-day low-dose primaquine. Preliminary results for the first two aims were presented, while for the last aim, an interactive exercise with participants was conducted to inform outputs of interest that can best inform policymaking.

Meeting participants were interested in more detailed cost data for 7-day high-dose primaquine and tafenoquine—specifically the cost of implementation, which was not included in our initial estimates. For instance, key stakeholders requested that supply chain or distribution costs at a national and subnational level be included in the analysis, in addition to costs of training, referral, and follow-up, and the costs for providing treatments at the health centre as compared to provision in the community. These suggestions were in addition to the variables currently included in the cost analysis, such as the reduced opportunity costs for patients, who would normally have to stay home for the duration of treatment, and the reduced health centre workload.

While Cambodia has already implemented G6PD testing at the health centre level, analysers were purchased before the STANDARD G6PD test was WHO prequalified. There were concerns that supplies such as test strips produced for the new WHO prequalified version of the STANDARD G6PD test would not be compatible with the analysers currently used. If not compatible, then new analysers would need to be purchased, requiring additional funding commitments [54]. In a post-meeting communication with the manufacturer, the control reagents and test strips were confirmed to be compatible between the WHO prequalified versions and non-prequalified versions (personal communication, Roxana Jung, SD Biosensor).

**Fig. 1:**
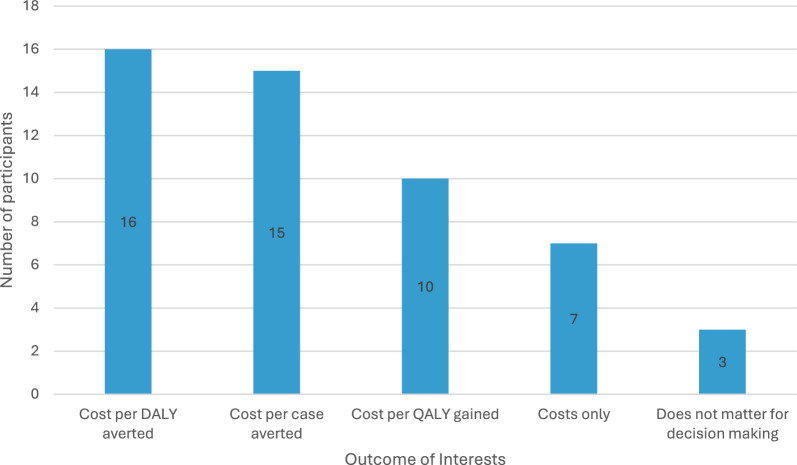
Outcomes of interest to meeting attendees for the cost-effectiveness analysis. *DALY* disability-adjusted life-year, *QALY* quality-adjusted life-year

The EFFORT study collected EQ-5D surveys alongside the trial, which will enable the calculation of quality-adjusted life-year (QALYs) lost per malaria episode. To date, the malaria economic evaluation literature has used disability-adjusted life-years (DALYs) or malaria cases averted for the outcome measure [55, 56]. DALYs are generic measures that rely on disability weights estimated by the Global Burden of Disease Study [57] and are commonly used in low- and middle-income settings where tariffs to convert EQ-5D into index scores that reflect how the population values health states are not available. While Cambodia does not have a tariff, Thailand’s tariff could be used [58]. The outcome measure of cases averted is useful for decision-making within the malaria control programme; however, QALYs and DALYs enable comparison with other disease areas (e.g., cancer treatment). Participants were especially interested in cost per DALY averted and cost per case averted (Fig. 1). Accordingly, the planned cost-effectiveness analysis will present the cost per DALY averted as the primary outcome with a sensitivity analysis to examine the results for cost per case averted.

A model-based cost-effectiveness analysis including current policy could incorporate data from outside the EFFORT trial and would potentially allow for more policy-relevant information. To guide the analysis plan, attendees were presented with a range of questions detailed in Table 1. Participants overwhelmingly expressed support for tafenoquine throughout the discussion, but there were mixed answers to what the most important comparators should be for the cost-effectiveness analysis, suggesting that a comparison of all treatment options is still warranted. These findings are in line with the desire for flexible and all-encompassing treatment guidelines.

For their preference for an analytical approach, meeting attendees selected a transmission model framework as the more helpful methodology, highlighting the perceived value of incorporating transmission dynamics as opposed to a decision tree model, which would only evaluate the impact on individual patients. In view of the small number of cases, it was decided that sufficient data were not available to appropriately calibrate a transmission model for this analysis. Accordingly, a decision tree will be used for the cost-effectiveness analysis instead.

## Conclusion

Stakeholders agreed that EFFORT study results should be incorporated into Cambodia’s National Treatment Guidelines, as the Ministry of Health has declared that *“we must use all means to eliminate malaria”.* Stakeholders preferred a single-dose tafenoquine over 7-day high-dose primaquine; however, there are feasibility challenges that need to be considered, including its off-label use in combination with ACT and related procurement challenges. Stakeholders agreed that treatment guidelines should be as inclusive and flexible as possible, giving policymakers the options to implement the best available treatments once feasibility considerations have been addressed. In addition to feasibility considerations, part of the decision-making process will be informed by cost-effectiveness analysis. The demand for shorter treatment options exists despite the changing malaria burden in Cambodia, as they present added value for reaching hard-to-reach high-risk populations, outbreak responses, and preventing reintroduction.

## Data Availability

No datasets were generated or analysed during the current study.

## Abbreviations

ACT: Artemisinin-based combination therapy
ASMQ: Artemisinin-mefloquine
CHAI: Clinton health access initiative
CNM: Cambodia’s National Center for Parasitology, Entomology and Malaria Control
DALY: Disability-adjusted life-years
DHA-piperaquine: Dihydroartemisinin-piperaquine
G6PD: Glucose-6-phosphate dehydrogenase
GMS: Greater Mekong Subregion
MMV: Medicines for malaria venture
QUALY: Quality-adjusted life-year
UNOPS: United Nations Office for Project Services
PMI: President Malaria Initiative
WHO: World Health Organization
